# The Inflammatory Profile of CTEPH-Derived Endothelial Cells Is a Possible Driver of Disease Progression

**DOI:** 10.3390/cells10040737

**Published:** 2021-03-26

**Authors:** Valérie F. E. D. Smolders, Kirsten Lodder, Cristina Rodríguez, Olga Tura-Ceide, Joan Albert Barberà, J. Wouter Jukema, Paul H. A. Quax, Marie José Goumans, Kondababu Kurakula

**Affiliations:** 1 Department of Surgery, Einthoven Laboratory for Experimental Vascular Medicine, Leiden University Medical Center, 2300 RC Leiden, The Netherlands; v.f.e.d.smolders@lumc.nl (V.F.E.D.S.); P.H.A.Quax@lumc.nl (P.H.A.Q.); 2 Department of Cell and Chemical Biology, Laboratory for CardioVascular Cell Biology, Leiden University Medical Center, 2300 RC Leiden, The Netherlands; K.Lodder@lumc.nl (K.L.); M.J.T.H.Goumans@lumc.nl (M.J.G.); 3 Department of Pulmonary Medicine, Hospital Clínic-Institut d’Investigacions Biomèdiques August Pi i Sunyer (IDIBAPS), University of Barcelona, 08036 Barcelona, Spain; cristina.rodriguezgut@gmail.com (C.R.); olgaturac@gmail.com (O.T.-C.); JBARBERA@clinic.cat (J.A.B.); 4 Department of Pulmonary Medicine, Dr. Josep Trueta University Hospital de Girona, Santa Caterina Hospital de Salt and the Girona Biomedical Research Institut (IDIBGI), 17190 Girona, Spain; 5 Biomedical Research Networking Centre on Respiratory Diseases (CIBERES), 28029 Madrid, Spain; 6 Department of Cardiology, Leiden University Medical Center, 2300 RC Leiden, The Netherlands; j.w.jukema@lumc.nl

**Keywords:** chronic thromboembolic pulmonary hypertension, inflammation, nuclear factor-κB signaling, endothelial dysfunction

## Abstract

Chronic thromboembolic pulmonary hypertension (CTEPH) is a form of pulmonary hypertension characterized by the presence of fibrotic intraluminal thrombi and causing obliteration of the pulmonary arteries. Although both endothelial cell (EC) dysfunction and inflammation are linked to CTEPH pathogenesis, regulation of the basal inflammatory response of ECs in CTEPH is not fully understood. Therefore, in the present study, we investigated the role of the nuclear factor (NF)-κB pro-inflammatory signaling pathway in ECs in CTEPH under basal conditions. Basal mRNA levels of interleukin (IL)-8, IL-1β, monocyte chemoattractant protein-1 (MCP-1), C-C motif chemokine ligand 5 (CCL5), and vascular cell adhesion molecule-1 (VCAM-1) were upregulated in CTEPH-ECs compared to the control cells. To assess the involvement of NF-κB signaling in basal inflammatory activation, CTEPH-ECs were incubated with the NF-κB inhibitor Bay 11-7085. The increase in pro-inflammatory cytokines was abolished when cells were incubated with the NF-κB inhibitor. To determine if NF-κB was indeed activated, we stained pulmonary endarterectomy (PEA) specimens from CTEPH patients and ECs isolated from PEA specimens for phospho-NF-κB-P65 and found that especially the vessels within the thrombus and CTEPH-ECs are positive for phospho-NF-κB-P65. In summary, we show that CTEPH-ECs have a pro-inflammatory status under basal conditions, and blocking NF-κB signaling reduces the production of inflammatory factors in CTEPH-ECs. Therefore, our results show that the increased basal pro-inflammatory status of CTEPH-ECs is, at least partially, regulated through activation of NF-κB signaling and potentially contributes to the pathophysiology and progression of CTEPH.

## 1. Introduction

Chronic thromboembolic pulmonary hypertension (CTEPH) is a severe cause of pulmonary hypertension (PH) resulting from unresolved pulmonary emboli (PE) that obstruct the main pulmonary arteries. In addition, vascular remodeling of muscular pulmonary arteries, similar to the arteriopathy observed in pulmonary arterial hypertension (PAH), is found in CTEPH [1,2,3]. CTEPH patients, without medical intervention, have a poor prognosis, with a five-year survival rate between 10 and 30% depending on the mean pulmonary artery pressure [4]. Pulmonary endarterectomy (PEA) to remove fibrotic organized clots from pulmonary arteries is the gold-standard treatment for eligible patients with CTEPH and significantly improves patients’ survival and hemodynamics [5]. The invasiveness of PEA surgery together with the insufficient effects of additional treatment options for inoperable patients and for patients with recurrent/persistent PH (up to 35%) after PEA indicate the importance of resolving new, potentially curative targets in CTEPH pathogenesis [4]. Balloon pulmonary angioplasty is emerging as a treatment option for inoperable CTEPH, but knowledge of the long-term effects on hemodynamics and patient survival remains limited [6].

Only 75% of patients with CTEPH have a history of symptomatic acute PE [7]. To date, the molecular and cellular mechanisms behind the lack of thrombus resolution and vascular remodeling that result in CTEPH remain unclear. The frequently observed remodeling in non-occluded arteries and small pulmonary arteries, similar to that observed in PAH, supports the presence of endothelial dysfunction in CTEPH pathogenesis [2]. Studies have reported the involvement of endothelial cells (ECs) in the process of thrombi organization and remodeling of surrounding pulmonary arteries through impaired angiogenesis, changes in EC function, and increased production of inflammatory cytokines and adhesion molecules [8,9,10,11,12,13]. The pulmonary endothelium is an important interface between the circulating blood and the underlying tissues; through the production and release of cytokines, chemokines, and adhesion molecules, it controls inflammatory cell adhesion and trafficking [14].

The expression and production of inflammatory mediators in the endothelium are controlled by nuclear factor (NF)-κB, a central regulator of inflammation [15,16]. Activated NF-κB translocates into the nucleus to promote the expression of target genes, such as tumor necrosis factor alpha (TNFα), interleukin-1-beta (IL-1β), IL-8, monocyte chemoattractant protein- 1 (MCP-1), vascular cell adhesion molecule-1 (VCAM-1), and intracellular adhesion molecule-1 (ICAM-1), among others [15]. Several of these endothelial-derived inflammatory factors have been shown to influence cell survival, proliferation, and the migration of cells within the vascular wall, thereby contributing to vascular remodeling [11,16,17,18].

Resting ECs suppress the transcription of pro-inflammatory factors to maintain vessel homeostasis. Upon activation, the resulting expression of inflammatory cytokines and adhesion molecules plays a pivotal role in modulating inflammation [19]. A balanced production of cytokines is key in order to maintain an intact endothelium and healthy pulmonary vessels [20]. In the context of PH, a pro-inflammatory component has been suggested and observed in both pulmonary arterial hypertension (PAH) and CTEPH [21,22,23,24,25]. However, most knowledge has been obtained through studies in which ECs are stimulated by a pro-inflammatory stimulus. Wynants et al. (2013) showed that the NF-κB pathway is involved in C-reactive protein-induced effects on ECs in CTEPH [21]. To gain insights into the inflammatory phenotype of unstimulated ECs, in this manuscript, we examine basal gene expression of inflammatory factors and use inhibitory small molecules such as Bay 11-7085 [26] to study the involvement of the NF-κB pathway in the activation of inflammatory pathways in CTEPH-ECs.

## 2. Materials and Methods

### 2.1. Study Population and Samples Collected

This study included CTEPH-ECs derived from 8 different endarterectomy specimens from patients with CTEPH who underwent pulmonary endarterectomy at the Hospital Clínic of Barcelona, Spain. The study was conducted in accordance with the Declaration of Helsinki and approved by the Institutional Committee on Human Research (Hospital Clínic of Barcelona ethics committee (HCB/2018/0837 and HCB/2018/0434)). The study was approved by the Institutional Ethics Committee of the Hospital Clínic of Barcelona and written informed consent was obtained from all patients. All patients were diagnosed according to the 2015 ESC/ERS Guidelines [27].

### 2.2. Pulmonary Endothelial Cell Isolation and Culture

ECs isolated from endarterectomy specimens, referred to as CTEPH-ECs, were cultured as previously described [28]. In short, ECs were plated onto 0.1% gelatin-coated wells and grown in an endothelium cell medium (ScienceCell Research Laboratories) supplemented with endothelial cell growth supplement, 5% fetal bovine serum (FBS), and Penicillin/Streptomycin solution (ScienceCell Research Laboratories). The cell phenotype was characterized by staining the cells with antibodies against a panel of endothelial and smooth muscle cell-specific markers, including endothelial nitric oxide synthase (eNOS) and alpha smooth muscle Actin (αSMA) [28]. The patient characteristics are presented in Table 1. Three different batches of human pulmonary artery endothelial cells (HPAECs) (Lonza, CC-2530) were used as control cells.

### 2.3. Gene Expression Analysis

The levels of IL-8, MCP-1, C-C motif chemokine ligand 5 (CCL5), IL-1β, ICAM-1, and VCAM-1 were measured from CTEPH-ECs cultured in low-serum conditions (endothelial cell medium with 0.1% FBS) by real-time quantitative PCR (*n* = 8 per group). Bay 11-7085 (Calbiochem, Millipore, North Holland, the Netherlands), a potent NF-κB inhibitor, was applied at 1μM final concentration. Treatments were performed in an endothelial cell medium with 0.1% FBS, and stimuli were provided for 6 h. Total RNA was extracted using the ReliaPrep™ RNA Cell Miniprep system (Promega, Leiden, the Netherlands) and concentrations were determined by spectrophotometry. Reverse transcription was performed using a RevertAid First Strand cDNA Synthesis Kit (ThermoFisher Scientific, Leiden, the Netherlands). For qRT-PCR, a QuantiTect^®^ SYBR^®^ Green PCR Kit (Qiagen, Venlo, the Netherlands) and specific primers were used on the ViiA7 Real-Time PCR system (Applied Biosystems, Bleiswijk, the Netherlands). Relative quantification was calculated by normalizing the threshold cycle (Ct) of the gene of interest to the Ct of an endogenous control (TATA-Box Binding Protein (TBP) and acidic ribosomal protein (ARP)) in the same sample, using the comparative ΔΔCt method. All primers were produced by Invitrogen, and the primer sequences can be found in Table 2.

### 2.4. Immunostaining

Paraffin-embedded sections (5 μm) of pulmonary endarterectomy specimens from CTEPH patients (*n* = 8) were incubated overnight at 4 °C with antibodies against phospho-NF-κB-P65 (pP65) (rabbit anti-phospho-p65, 1:100; cat#11260, SAB Biotech, Baltimore, MD, USA,) and platelet endothelial cell adhesion molecules (goat anti-CD31, 1:1000; R&D Systems cat#AF3628, Minneapolis, Minnesota, USA). Sections were then incubated with anti-rabbit Alexa Fluor 555 (Invitrogen, cat#A31572, Leiden, the Netherlands) or anti-goat Alexa Fluor 647 (Invitrogen, cat#A21447, Leiden, the Netherlands) secondary antibodies for 2 h. Nuclei were counterstained using Hoechst 34580 (1:800; Sigma-Aldrich, cat#63493, Zwijndrecht, the Netherlands). Sections were imaged using a slide scanner (3DHistech Pannoramic 250). Colocalization analysis was performed with JACop, an ImageJ plugin [29]. We counted all of the vessels in every patient (*n* = 8) using whole scanned images and found that the number of CD31+ve vessels in each patient was highly variable. Importantly, some patients’ sections did not contain any vessels, which made it difficult to combine the data from all patients. However, almost all of the vessels contained pP65+ve cells, suggesting activation of the NF-κB pathway in the PEA tissue of CTEPH patients. Lung tissue from chronic obstructive pulmonary disease (COPD, (*n* = 4) and healthy subjects (*n* = 2) and aortic tissue from bicuspid aortic valve disease patients (*n* = 3) were used for validation of the phospho-NF-κB-P65 antibody. Furthermore, paraffin-embedded sections from both CTEPH and control subjects were incubated with only secondary antibody as a negative control for the phospho-NF-κB-P65 antibody.

CTEPH-ECs and HPAECs were seeded at 1 × 10^5^ cells/mL in 24-well plates pre-coated with 1% gelatin and covered with glass cover slides and allowed to grow in a complete endothelial cell medium. After 48 h, the cells were incubated in an endothelial cell medium with 0.1% FBS. Next, the cells were washed with cold PBS and fixated with 4% paraformaldehyde. The cells were then permeabilized with PBS/0.25% Triton and blocked with PBS/0.1% Triton/10% FBS. Next, the slides were incubated at 4 °C overnight with antibodies against phospho-P65 (rabbit anti-phospho-P65 1:100; cat#11260, SAB Biotech, Baltimore, MD, USA) and CD31 (mouse anti-CD31 1:250; Dako, cat#M0823, Santa Clara, CA, USA). The slides were then incubated with anti-rabbit Alexa Fluor 488 (Invitrogen, cat#A21206, Leiden, the Netherlands) or anti-mouse Alexa Fluor 647 (Invitrogen, cat#A21447, Leiden, the Netherlands) secondary antibodies for 1 h. The nuclei were counterstained with DAPI (ProLong™ Gold Antifade Mountant with DAPI, Invitrogen, cat#P36931, Leiden, the Netherlands). The slides were imaged using a Leica DM6B microscope and the mean fluorescence intensity was quantified using ImageJ software.

### 2.5. Statistical Analysis

Results are described as mean ± standard deviation and were compared using unpaired *t*-test (immunofluorescence analysis) or unpaired *t*-test with Welch’s correction (gene expression analysis). Statistical analyses were performed using GraphPad Prism version 8. *p*-values ≤ 0.05 were considered statistically significant.

## 3. Results

### 3.1. Basal Inflammatory Gene Expression in CTEPH-ECs and PAH-ECs

**Figure 1:**
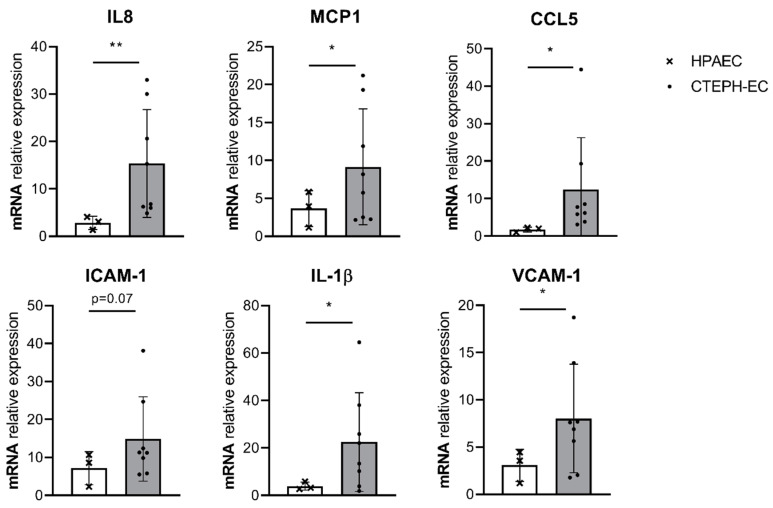
**Basal inflammatory gene expression in cultured CTEPH endothelial cells (ECs).** mRNA expression levels of interleukin (IL)-8, IL-1β, monocyte chemoattractant protein- 1 (MCP-1), C-C motif chemokine ligand 5 (CCL5), vascular cell adhesion molecule-1 (VCAM-1), and intracellular adhesion molecule-1 (ICAM-1) were found increased under basal conditions (0.1 serum) in CTEPH-ECs compared to human pulmonary artery endothelial cells (HPAECs). CTEPH-EC, *n* = 8 donors; HPAEC, *n* = 3 donors; unpaired *t*-test with Welch’s correction, *p* < 0.05 = *, data are expressed as mean ± SD.

A controlled release of inflammatory cytokines is essential to maintain a quiescent endothelium that prevents disease development. We determined whether the basal gene expression of inflammatory cytokines is disturbed in CTEPH-ECs, as a potential contributor to disease pathology. mRNA expression levels of IL-8 and MCP-1 showed a 5.5-fold (*p* = 0.009) and a 2.5-fold (*p* = 0.05) increase, respectively, compared to control cells. CCL5 showed a seven-fold increase (*p* = 0.03) in mRNA expression levels compared to control cells. ICAM-1 showed a trend towards increased levels in CTEPH-ECs but did not reach significance (*p* = 0.07). IL-1β showed a six-fold increase (*p* = 0.02) in mRNA levels compared to HPAECs. Finally, mRNA levels of VCAM-1 showed a three-fold (*p* = 0.03) increase in CTEPH-ECs compared to control cells (Figure 1).

### 3.2. Fluorescence Staining of Phospho-P65 in CTEPH-Ecs

**Figure 2:**
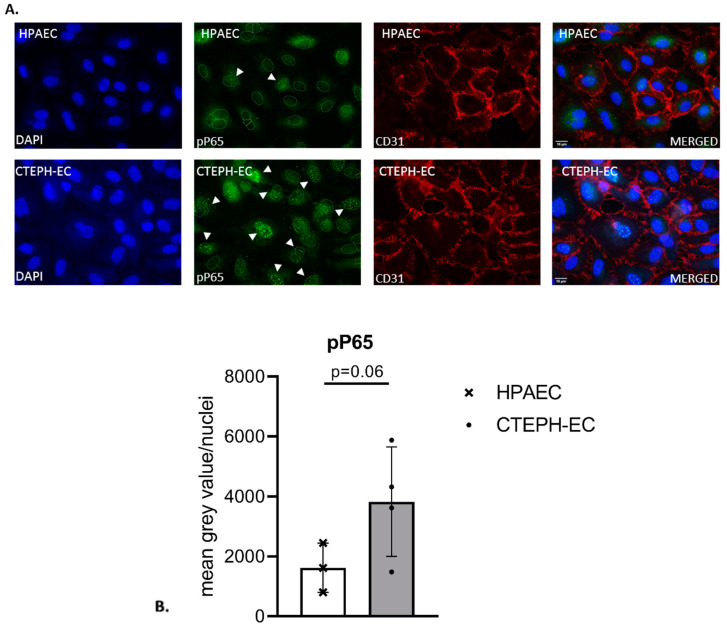
**Fluorescence staining of phospho-P65 in cultured ECs.** (**A**) HPAECs (top) and CTEPH-ECs (bottom) were stained for endothelial marker CD31/PECAM (red) and phospho-NF-κB-P65 (pP65; green). Both ECs showed the presence of CD31/PECAM and nuclear pP65 staining (indicated by the white arrows). Nuclei were counterstained with DAPI; scale bar, 10 µm. (**B**) The presence of nuclear pP65 was quantified in both CTEPH-ECs and HPAECs. CTEPH-ECs showed a 2.4-fold higher presence of nuclear pP65 compared to control cells (*p* = 0.06) (unpaired *t*-test); CTEPH-ECs, *n* = 4 donors; HPAECs, *n* = 3 donors. Data are expressed as mean ± SD.

**Figure 3:**
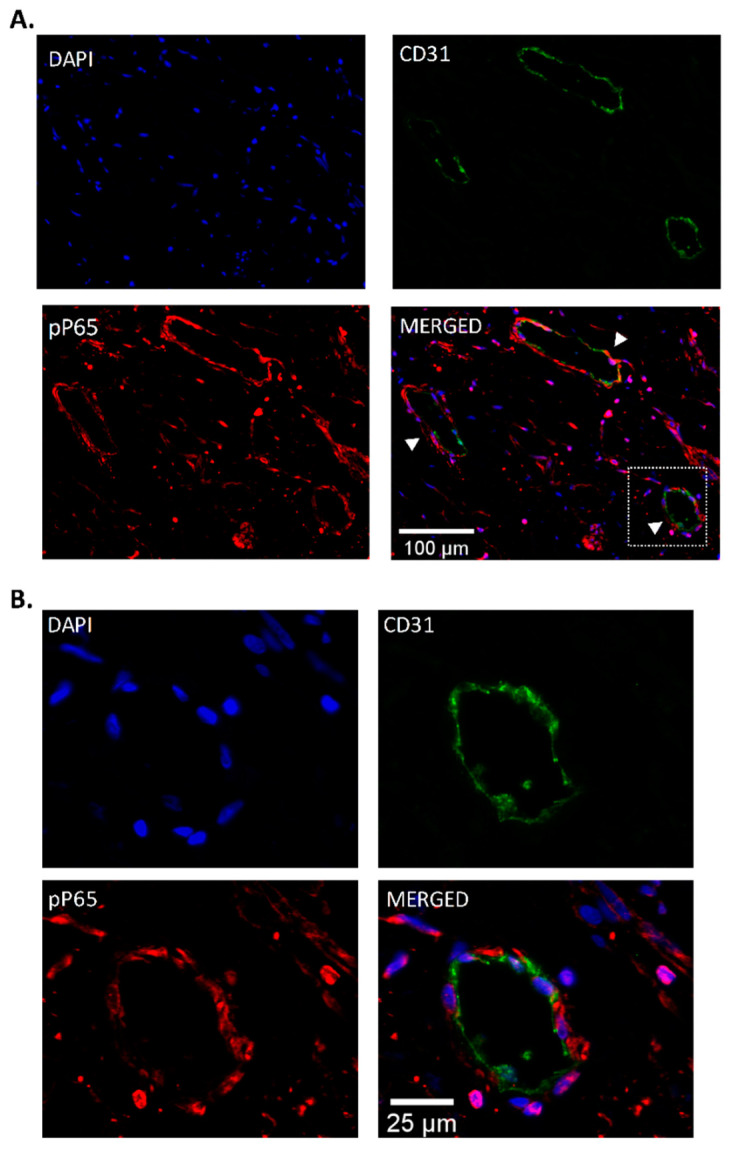
**Pulmonary endarterectomy (PEA) immunofluorescence.** (**A**) Representative images showing the localization of phospho-NF-κB-P65 (pP65) in vessels in endarterectomy specimens from patients with CTEPH (*n* = 8), using double labeling with CD31/PECAM (green) and pP65 (red). (**B**) pP65 immunoreactivity was observed in endothelial cells from vessels within the thrombus (magenta, indicated by the white arrows). Nuclei were counterstained with DAPI (blue). Scale bar, 100 μm (panel (**A**)) and 25 μm (panel (**B**)).

Activation of the expression of inflammatory cytokines is often the result of NF-κB signaling. Therefore, we studied NF-κB activation in CTEPH-ECs by monitoring nuclear translocation of the p65 subunit by immunofluorescence. Cultured CTEPH-ECs, isolated from four different pulmonary endarterectomy specimens and three different control HPAECs, were stained for CD31/PECAM and pP65. Both CTEPH-ECs and HPAECs were positive for the endothelial marker CD31/PECAM (Figure 2A). The number of cells showing nuclear anti-p65 was determined from three randomly selected areas. CTEPH-ECs and HPAECs showed a positive fluorescence signal for pP65, which was mainly found within DAPI-positive nuclei (Figure 3A). The amount of nuclear translocation of the pP65 subunit was determined by quantifying the intensity of the fluorescence signal inside the nuclei, and CTEPH-ECs showed a trend towards a 2.4-fold higher (*p* = 0.06) nuclear signal intensity compared to the control cells (Figure 2B).

### 3.3. Endothelial Localization of Phospho-P65 in CTEPH Specimen

Based on these in vitro results, pulmonary endarterectomy specimens from CTEPH patients were stained for the presence of phospho-NF-κB-P65 (pP65) and CD31/PECAM. Thrombus vessels present in the tissue stained positive for CD31/PECAM. Interestingly, in these areas, pP65 was shown to colocalize with CD31/PECAM, indicating the presence of pP65 ECs lining vessels within the thrombus. Cells positive for pP65, but negative for CD31/PECAM, were also observed throughout the tissue. These cells are most likely infiltrating inflammatory cells (Figure 3A,B).

### 3.4. Effect of NF-κB Inhibition in CTEPH-ECs

**Figure 4:**
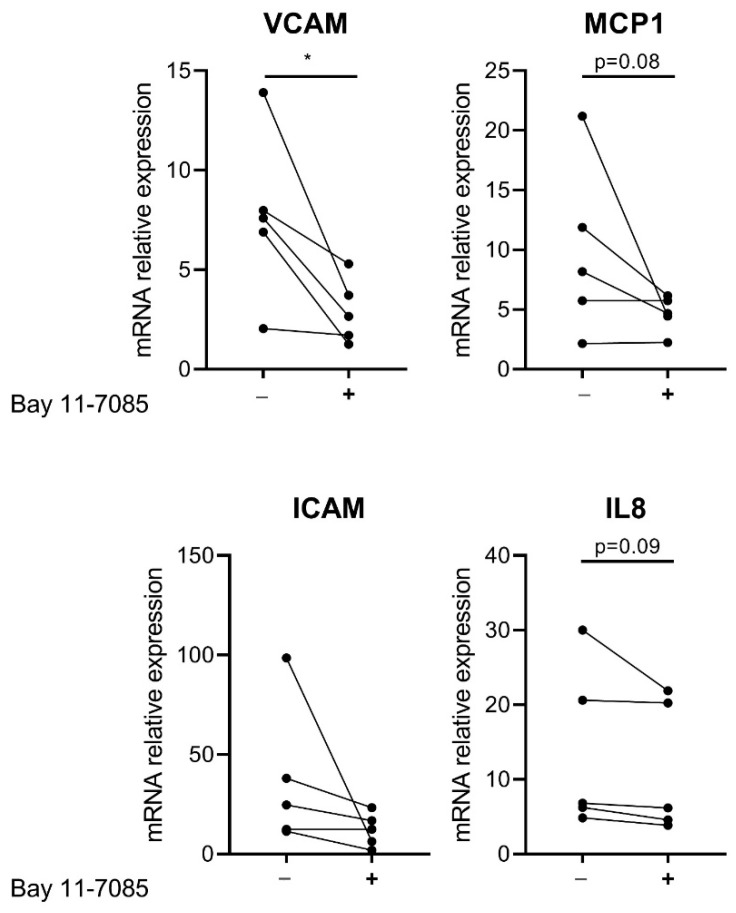
**Effect of NF-κB inhibition on CTEPH-ECs**. Cultured CTEPH-ECs were incubated with the NF-κB inhibitor Bay 11-7085 at a final concentration of 1 µM. mRNA levels of VCAM-1 were significantly reduced in CTEPH-ECs after treatment with Bay 11-7085. mRNA levels of ICAM-1 and MCP-1 showed a trend towards reduction after Bay 11-7085 incubation. IL-8 mRNA expression levels were found to be similar between the CTEPH-ECs incubated with Bay 11-7085 and those without. CTEPH-ECs, *n* = 5 donors; unpaired *t*-test, *p* < 0.05 = *, data are expressed as mean ± SD.

In order to confirm that the increased basal gene expression of inflammatory factors is due to increased NF-κB activity in CTEPH-ECs, cells were incubated with 1 µM Bay 11-7085, an inhibitor of NF-κB through inhibition of IκB degradation. mRNA levels of VCAM-1 showed a 1.6-fold reduction (*p* = 0.02) in CTEPH-ECs compared to the control condition without the inhibitor. ICAM-1 and MCP-1 showed a tendency towards reduction after incubation with Bay 11-7085 but did not reach significance (*p* = 0.09 and *p* = 0.08, respectively). mRNA levels of IL-8 were found to be unchanged in CTEPH-ECs after incubation with Bay 11-7085 compared to the control condition (Figure 4).

## 4. Discussion

In this study, we showed that the basal gene expression of the inflammatory factors downstream of the NF-κB signaling pathway such as IL-8, IL-1β, CCL5, MCP-1, and VCAM-1, was increased in CTEPH-ECs. The increase in the expression of inflammatory factors was accompanied by an increased nuclear localization of pP65 in the cultured CTEPH-ECs, indicating the presence of more active NF-κB signaling in CTEPH-ECs compared to the control HPAECs. Furthermore, pP65-positive thrombus vessels were observed in CTEPH PEA specimens. Lastly, CTEPH-ECs incubated with the NF-κB inhibitor Bay 11-7085 showed a decrease in the expression of VCAM-1, ICAM-1, and MCP-1.

Although a pro-inflammatory phenotype in CTEPH is to be expected [22], we showed that, in contrast to previous studies [21], CTEPH-ECs also present, under basal conditions, a pro-inflammatory status. Only a few studies have been performed to better understand the regulatory pathways of inflammation in CTEPH pathogenesis. Ataam et al. recently reported that increased ICAM-1 contributes to EC dysfunction in CTEPH [12]. In the present study, we showed that cultured CTEPH-ECs have increased nuclear phospho-p65 under basal conditions compared to control cells. In addition to phosphorylation and degradation of inhibitory protein IκBs, NF-κB activation also involves phosphorylation of the P65 subunit. This phosphorylation is an important event to enhance the transcriptional capacity of DNA-bound NF-κB and activates the transcription of the key targets VCAM-1, ICAM-1, and MCP-1 in ECs [30,31,32]. Therefore, our results indicate that the observed increase in phosphorylation of the P65 subunit is key to the elevated basal expression of inflammatory cytokines in CTEPH-ECs. Several inflammation-related diseases such as cancer, atherosclerosis, restenosis, and asthma have been associated with increased activation of NF-κB and expression of its downstream mediators [33,34,35,36,37]. Based on the increase in NF-κB activation observed, we hypothesized that inhibition of NF-κB signaling could reverse the basal pro-inflammatory profile in CTEPH-ECs. In the current study, we found that the inhibition of NF-κB signaling by blocking the phosphorylation of the NF-κB inhibitor IκB-α with the inhibitory small molecule Bay 11-7085 results in a reduced expression of the inflammatory cytokines VCAM-1, ICAM-1, and MCP-1 in CTEPH-ECs. These findings confirm that the increased inflammatory cytokines in CTEPH-ECs are, at least partially, regulated through NF-κB signaling. Cancers such as multiple myeloma, where NF-κB signaling plays a significant role in the pathogenesis, have been successfully treated with drugs that have NF-κB as their primary or secondary target [38], suggesting that inflammation is a potential target in the search for novel pharmacological interventions to prevent disease progression in CTEPH.

Limitations of this study: Due to the high heterogeneity among the CTEP-ECs (*n* = 8) used in this study, more ECs from CTEPH patients should be included to further strengthen the current findings. Another potential limitation is the lack of validation of the NF-κB-dependent pP65 expression levels by Western blotting and the detection of pro-inflammatory cytokines by ELISAs. Therefore, future research should be warranted to investigate this signaling pathway in more detail using large number of samples.

Based on the results obtained in this study, we can conclude that CTEPH-ECs have a basal pro-inflammatory status, shown by the increased production of the inflammatory cytokines IL-8, MCP-1, IL-1β, CCL5, ICAM-1, and VCAM-1 under basal conditions. More importantly, we showed that this basal pro-inflammatory status observed in CTEPH-ECs is, at least partially, regulated through NF-κB signaling, and blocking NF-κB activation might be an important target in CTEPH to prevent disease progression or recurrent PH.

## Figures and Tables

**Table 1:** Clinical features and hemodynamic parameters.

	CTEPH (*n* = 8)
Female/male	5/3
Age years	63.15 ± 10.88
BMI kg·m^−2^	25.97 ± 4.35
mPAP mmHg	42.13 ± 9.52
PVR dyn·s·m^−5^	706.75 ± 230.07
PAOP mmHg	10.25 ± 3.77
Cardiac index L·min^−1^·m^−2^	2.23 ± 0.61
Right atrial pressure mmHg	9.38 ± 4.63
SvO2 %	59.50 ± 7.86
6MWD m	398.13 ± 102.02
BNP pg·mL^−1^	209.54 ± 360.12
History of VTE	1/7
WHO FC	
I	0
II	2
III	6

Data are presented as n or mean ± SD. CTEPH: chronic thromboembolic pulmonary hypertension; BMI: body mass index; mPAP: mean pulmonary artery pressure; PVR: pulmonary vascular resistance; PAOP: pulmonary artery occlusion pressure; SvO2: mixed venous oxygen blood saturation; 6MWD: 6-min walking distance; BNP: brain natriuretic peptide; VTE: venous thromboembolism; WHO FC: World Health Organization functional class.

**Table 2:** Primer sequences.

Gene Name	Forward Primer (5′-3′)	Reverse Primer (5′-3′)
IL-8	CTGGCCGTGGCTCTCTTG	CTTGGCAAAACTGCACCTTCA
MCP-1	CTGTGCCTGCTGCTCATAG	AGCTTCTTTGGGACACTTGC
CCL5	GCATCTGCCTCCCCATATTC	AGTGGGCGGGCAATGTAG
IL-1β	CGAATCTCCGACCACCACTAC	TCCATGGCCACAACAACTGA
ICAM	CTGCAGACAGTGACCATC	GTCCAGTTTCCCGGACAA
VCAM	CAGGCTGGAAGAAGCAGA	GGCCTTTCGGATGGTATAGG
ARP	CACCATTGAAATCCTGAGTGATGT	TGACCAGCCGAAAGGAGAAG
TBP	TGGAAAAGTTGTATTAACAGGTGCT	GCAAGGGTACATGAGAGCCA

## Data Availability

The data presented in this study are available on request from the corresponding author.
